# Diabetes Epidemiology Among Adults in Port-au-Prince, Haiti: A Cross-Sectional Study

**DOI:** 10.3389/fendo.2022.841675

**Published:** 2022-02-24

**Authors:** Rodney Sufra, Jean Lookens Pierre, Eliezer Dade, Vanessa Rouzier, Alexandra Apollon, Stephano St Preux, Fabiola Préval, Joseph Inddy, Miranda Metz, Olga Tymejczyk, Denis Nash, Rodolphe Malebranche, Marie Deschamps, Jean W. Pape, Marcus D. Goncalves, Margaret L. McNairy, Lily D. Yan

**Affiliations:** ^1^Haitian Group for the Study of Kaposi’s Sarcoma and Opportunistic Infections (GHESKIO), Port-au-Prince, Haiti; ^2^Center for Global Health, Department of Medicine, Weill Cornell Medicine, New York, NY, United States; ^3^Institute for Implementation Science in Population Health, City University of New York, New York, NY, United States; ^4^Collège Haïtien de Cardiologie, Port-au-Prince, Haiti; ^5^Medicine and Pharmacology, Université d’État d’Haïti, Port-au-Prince, Haiti; ^6^Division of Endocrinology, Diabetes, and Metabolism, Department of Medicine, Weill Cornell Medicine, New York, NY, United States; ^7^Division of General Internal Medicine, Department of Medicine, Weill Cornell Medicine, New York, NY, United States

**Keywords:** diabetes mellitus, Haiti, Caribbean region, epidemiology, cardiovascular risk factor

## Abstract

**Introduction:**

Diabetes mellitus is a chronic noncommunicable disease associated with death and major disability, with increasing prevalence in low- and middle-income countries. There is limited population-based data about diabetes in Haiti. The objective of this study was to assess the prevalence of diabetes and associated factors among adults in Port-au-Prince, Haiti using a population-based cohort.

**Methods:**

This study analyzes cross-sectional enrollment data from the population-based Haiti Cardiovascular Disease Cohort Study, conducted using multistage sampling with global positioning system waypoints in census blocks in the metropolitan area of Port-au-Prince, Haiti. A total of 3,005 adults ≥18 years old were enrolled from March 2019 to August 2021. We collected socio-demographic data, health-related behaviors, and clinical data using standardized questionnaires. Diabetes was defined as any of the following criteria: enrollment fasting glucose value ≥ 126 mg/dL or non-fasting glucose ≥ 200 mg/dL, patient self-report of taking diabetes medications, or study physician diagnosis of diabetes based on clinical evaluation.

**Results:**

Among 2985 (99.3%) with complete diabetes data, median age was 40 years, 58.1% were female, and 17.2% were obese. The prevalence of diabetes was 5.4% crude, and 5.2% age standardized. In unadjusted analysis, older age, higher body mass index (BMI), low physical activity, low education were associated with a higher odds of diabetes. After multivariable logistic regression, older age [60+ vs 18-29, Odds Ratio (OR)17.7, 95% CI 6.6 to 47.9] and higher BMI (obese vs normal/underweight, OR 2.7, 95% CI 1.7 to 4.4) remained statistically significantly associated with higher odds of diabetes.

**Conclusion:**

The prevalence of diabetes was relatively low among adults in Port-au-Prince, but much higher among certain groups (participants who were older and obese). The Haitian health system should be strengthened to prevent, diagnose, and treat diabetes among high-risk groups.

## Introduction

Diabetes mellitus (DM) is a chronic disease and major health issue associated with premature mortality and poor quality of life, accounting for 5 million deaths per year (1, 2). According to the 2019 Global Burden of Disease project, diabetes is the fifth leading cause of death and eighth leading cause of disability worldwide, with increasing prevalence in low-middle income countries (LMICs) (3). Among the 400 million people with diabetes, 4 out of 5 live in LMICs (3). In addition, LMICs have larger proportions of undiagnosed diabetes than high income countries (80-90% in sub-Saharan Africa vs 20-30% in Western Europe and North America) (3).

Black Caribbean populations may have distinct diabetes profiles compared to other people of African descent. While type 2 DM occurs across the entire range of body mass index (BMI) in African populations, DM is worse in obese individuals of Caribbean descent, and Caribbean individuals also have worse glycemic control, lower health literacy, and a higher rate of complications compared to Africans or African Americans (4). An analysis by the International Diabetes Federation estimated the age adjusted prevalence of diabetes to be 9.6% in the Caribbean, however these estimates are based on extrapolation from a small number of studies as many countries do not yet have representative primary data (5). In a population-representative survey in Barbados, 4.9% had diabetes by A1c and 3.5% had diabetes by fasting plasma glucose (FPG) (5). A 2002 study of diabetes in Haiti found an age-standardized prevalence of diabetes at 4.8% in men and 8.9% in women, although a lower response rate of 69% limited generalizability (6).

There is limited population-based data concerning the epidemiology of diabetes among the adult population in Haiti. A pilot study conducted in 2016 in four slum communities found 1% had a prior self-reported diagnosis of diabetes among a young adult population (7). The aim of the present study was to use a large population-based cohort of adults in Port-au-Prince to assess the prevalence of diabetes and prediabetes, and examine their relationship with sociodemographic variables and health behaviors.

## Materials and Methods

### Data Source/Study Population

We analyzed cross-sectional enrollment data within the longitudinal Haiti Cardiovascular Disease (CVD) cohort study to assess the prevalence of diabetes among adults in Port-au-Prince. This study enrolled 3,005 adults ≥ 18 years old living in the metropolitan area of Port-au-Prince. Participants were selected using multistage random sampling using Global Positioning System (GPS) waypoints within census blocks, with the number of waypoints per block proportional to the estimated size of the population. Our analytic population included all enrolled participants. Eligibility criteria for the study included an age of 18 or older, main residence in the Port-au-Prince study area (with no plans to move in the next 24 months), ability to give consent for study procedures and the willingness to be contacted in a new residence in the event of a move. Exclusion criteria included serious medical conditions or cognitive impairment preventing study participation, as judged by research physicians, or inability to speak and understand French or Creole. Participants with missing information on blood glucose values, self-report of taking diabetes medications, and physician diagnosis were excluded from our analysis (n=20).

The study was conducted at the Groupe Haïtien d’etude du Sarcome de Kaposi et des Infections opportunistes (GHESKIO), which is a large public health clinic established since 1982 in downtown Port-au-Prince. GHESKIO has operated continuously to provide clinical care and research on persons living with HIV since its origin, with expansion to other infections and chronic diseases, including CVD.

### Measurements and Outcomes

All data were collected during the enrollment survey. Sociodemographic data (age, sex, education, income) were collected using a standardized questionnaire. We classified the level of education in two groups: primary school or lower versus secondary school or higher. Daily income was measured in Haitian Gourdes and converted into two categories of ≤1 US Dollar (USD) versus >1 USD.

Health behaviors (smoking status, alcohol intake, physical activity, diet) were collected using standardized World Health Organization STEPwise Approach to NCD Risk Factor Surveillance instruments (8).

Smoking status was ascertained from questions asking if the participant ever smoked tobacco, and if they currently smoke tobacco. Alcohol intake was grouped into ≤1 drink daily or >1 drink daily. Physical activity was determined from questions asking whether the participant did vigorous work or recreational activity for > 75 minutes a week, or moderate work or recreational activity for > 150 minutes a week. Participants were categorized as “moderate-high” if they reported yes to moderate or vigorous activity. We categorized diet by the median daily servings of fruit or vegetable intake, and categorizing it by < 5 servings a day versus ≥5 servings a day (the recommended limit by the WHO) (8).

Lastly, sugar sweetened beverage or energy drink intake were ascertained from questions asking the number of days in a week the participant drank either.

Study physicians and nurses also collected vital signs and performed a clinical exam. Height and weight were used to calculate BMI (kg/m^2^) and determine if participants were obese, overweight, or normal. Laboratory data was also collected, including fasting and non-fasting venous plasma blood glucose using a Vitros 350 machine. Measurement of HbA1c is not routinely available at GHESKIO or in Haiti.

The primary study outcome was diabetes. We defined diabetes as any of the following criteria: enrollment fasting glucose value (FPG) ≥ 126 mg/dL or non-fasting glucose ≥ 200 mg/dL based on WHO definitions (9), patient self-report of taking diabetes medications, or study physician diagnosis of diabetes based on clinical evaluation. If a participant had a self-reported history of diabetes but was not on medication and had a normal glucose value, they were not categorized as having diabetes. Only one serum glucose measurement was obtained at enrollment. We were unable to differentiate between type I and type II diabetes.

Participants were classified as having prediabetes if they had an enrollment FPG ≥110 and < 126 mg/dL and they did not have diabetes (9). Finally, participants were considered normoglycemic if they did not meet the criteria for either diabetes or prediabetes.

### Statistical Analyses

For descriptive analyses, summary statistics including means, medians, interquartile ranges (IQR) and percentages were calculated for sociodemographic variables and health behaviors (smoking, alcohol, fruit/vegetable intake, sugar sweetened beverage intake, physical activity). Summary statistics were calculated across three groups: people with normoglycemia, people with prediabetes, and people with diabetes.

The prevalence of diabetes and standard error (SE) was calculated across the total sample, for both a crude prevalence and an age adjusted prevalence using the World WHO 2000-2025 Standard Population (10). The prevalence was then calculated stratified separately by age, sex, education, income, body mass index (BMI), fruit/vegetable intake, sugar sweetened beverage intake, and physical activity levels.

For inferential analyses, we used univariate and multivariable logistic regression to estimate the relationship between independent predictor variables (age, sex, education, income, smoking, alcohol, sugar sweetened beverage intake, physical activity) and the outcome of diabetes. Fruit/vegetable intake was not included as an independent predictor variable because there was almost no variation across the sample.

All analyses were conducted in R version 4.0.3.

### Ethics

This study was approved by institutional review boards at Weill Cornell Medicine and GHESKIO (record number 1803019037), with written participant consent.

## Results

A total of 3,005 participants were enrolled in this study, 20 were excluded due to missing data, for a final analytic data set at 2,985 (99.3%) (**Supplementary Figure 1**).

### Demographic Characteristics

Out of 2,985 participants, 2,778 had normoglycemia, 47 had prediabetes, and 160 had diabetes (**Table 1**). Among the 160 participants with diabetes, 49 (30.6%) had elevated glucose, 76 (47.5%) were on diabetes medications, 130 (81.2%) had a diagnosis of diabetes by a clinician, and 116 (72.5%) had a self-reported past medical history of diabetes.

**Table 1:** Sociodemographic and clinical characteristics for cohort of haitian adults.

	Total population (N = 2,985)	People with normo-glycemia (N = 2,778)	People with prediabetes (N = 47)	People with diabetes (N = 160)
Age, years	N (%)	N (%)	N (%)	N (%)
Median (IQR)	40 (27, 55)	39 (27, 53)	54 (40, 64)	54 (46, 62)
18-29	885 (29.6)	874 (31.5)	5 (10.6)	6 (3.8)
30-39	563 (18.9)	548 (19.7)	6 (12.8)	9 (5.6)
40-49	528 (17.7)	484 (17.4)	6 (12.8)	38 (23.8)
50-59	497 (16.6)	430 (15.5)	12 (25.5)	55 (34.4)
60+	512 (17.2)	442 (15.9)	18 (38.3)	52 (32.5)
**Female**	1734 (58.1)	1596 (57.5)	34 (72.3)	104 (65.0)
**Education**				
Primary or lower	1067 (35.8)	947 (34.2)	34 (72.3)	86 (53.8)
Secondary or higher	1912 (64.2)	1825 (65.8)	13 (27.7)	74 (46.2)
**Works for pay**	982 (33.0)	914 (33.0)	16 (34.0)	52 (32.5)
**Income (daily)**				
≤1 USD/day	2094 (70.3)	1951 (70.4)	34 (72.3)	109 (68.1)
>1 USD/day	885 (29.7)	821 (29.6)	13 (27.7)	51 (31.9)
**BMI, kg/m^2^**				
Underweight/Normal <24.9	1690 (56.7)	1627 (58.6)	15 (31.9)	48 (30.0)
Overweight 25.0-29.9	779 (26.1)	693 (25.0)	21 (44.7)	65 (40.6)
Obese ≥30.0	513 (17.2)	455 (16.4)	11 (23.4)	47 (29.4)
**Smoking status**				
Never/Former	2870 (96.4)	2671 (96.5)	46 (97.9)	153 (95.6)
Current	106 (3.6)	98 (3.5)	1 (2.1)	7 (4.4)
**Alcohol intake**				
≤1 drink a day	2860 (96.3)	2658 (96.1)	46 (97.9)	156 (98.7)
>1 drink a day	111 (3.7)	108 (3.9)	1 (2.1)	2 (1.3)
**Fruit/Vegetable intake**				
Median daily serving (IQR)	0.57 (0.29, 1.14)	0.57 (0.29, 1.14)	0.57 (0.29, 0.86)	0.71 (0.29, 1.29)
Range	0-14	0-14	0-4	0-5
<5 servings a day	2957 (99.3)	2750 (99.3)	47 (100.0)	160 (100.0)
≥5 servings a day	20 (0.7)	20 (0.7)	0 (0.0)	0 (0.0)
**Sugar sweetened beverage or energy drink intake**				
0-3 days a week	1047 (35.1)	936 (33.8)	25 (53.2)	86 (53.8)
4-7 days a week	1932 (64.9)	1836 (66.2)	22 (46.8)	74 (46.2)
**Physical activity**				
Low	1510 (50.8)	1384 (50.0)	25 (53.2)	101 (63.1)
Moderate-high	1464 (49.2)	1383 (50.0)	22 (46.8)	59 (36.9)
**Serum glucose measurements**				
Fasting, mean ± SD	94 ± 32	87 ± 8	115 ± 5	186 ± 79
Fasting range	46-416	46-109	110-125	84-416
Nonfasting, mean ± SD	94 ± 34	89 ± 13	–	204 ± 114
Nonfasting range	48-554	48-186	–	75-554

IQR, interquartile range.

Among people with prediabetes, the median age was 54 years old, 34 (72.3%) were female, 34 (72.3%) had a primary or lower education, and 34 (72.3%) had a daily income of ≤1 USD/day. For BMI, 11 (23.4%) were obese, for health behaviors 1 (2.1%) was a current smoker and 1 (2.1%) had >1 alcoholic drink a day. The fruit/vegetable intake was low, with a median daily serving of 0.57 (range 0 to 4). 22 (46.8%) people drank sugar sweetened beverages more than 4 days a week, and 25 (53.2%) had low physical activity. The mean fasting glucose value was 115 mg/dL.

Among people with diabetes, the median age was 54 years old, 104 (65.0%) were female, 86 (53.8%) had a primary or lower education, and 109 (68.1%) had a daily income of ≤1 USD/day. 47 (29.4%) were obese, 7 (4.4%) were current smokers, and 2 (1.3%) had >1 drink a day. The median daily servings of fruit or vegetables were 0.71 (range 0 to 5), and 74 (46.2%) people drank sugar sweetened beverages more than 4 days a week. 101 (63.1%) had low physical activity. Six participants were on insulin. The mean fasting glucose value was 186 mg/dL and mean non-fasting glucose value was 204 mg/dL.

### Diabetes Prevalence

There was a 5.4% crude prevalence of diabetes in our sample, and 5.2% age standardized prevalence of diabetes (**Table 2**).

**Table 2:** Prevalence of diabetes.

	Diabetes	
	Percent	SE
Age Standardized	5.2	0.4
Crude	5.4	0.4
**Age Categories (years)**		
18-29	0.7	0.3
30-39	1.6	0.5
40-49	7.2	1.1
50-59	11.1	1.4
60+	10.2	1.3
**Sex**		
Female	6	0.6
Male	4.5	0.6
**Education**		
Primary or lower	8.1	0.8
Secondary or higher	3.9	0.4
**Income**		
≤1 USD	5.2	0.5
>1 USD	5.8	0.8
**BMI**		
Underweight or Normal	2.8	0.4
Overweight	8.3	1
Obese	9.2	1.3
**Fruit/Vegetable intake**		
<5 servings a day	5.4	0.4
≥5 servings a day	0	0
**Sugar sweetened beverage intake**		
0-3 days a week	8.2	0.8
4-7 days a week	3.8	0.4
**Physical activity**		
Low	6.7	0.6
Moderate-high	4	0.5

**Figure 1:**
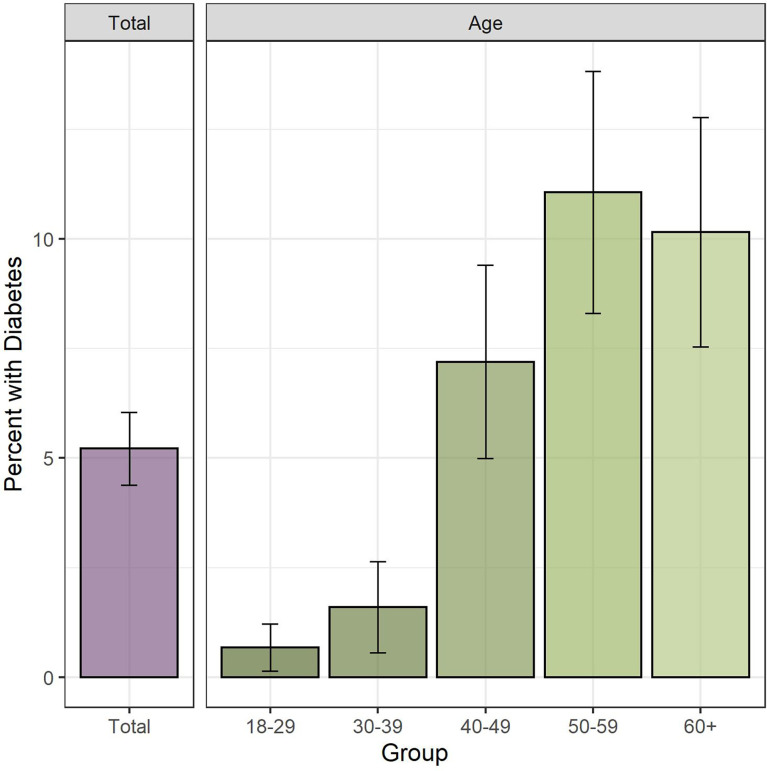
Diabetes prevalence overall and by age. Legend: Bar height indicates estimated diabetes prevalence, with 95% CI indicated by whiskers. Left panel shows total diabetes prevalence, right panel shows diabetes prevalence disaggregated by age categories.

**Figure 2:**
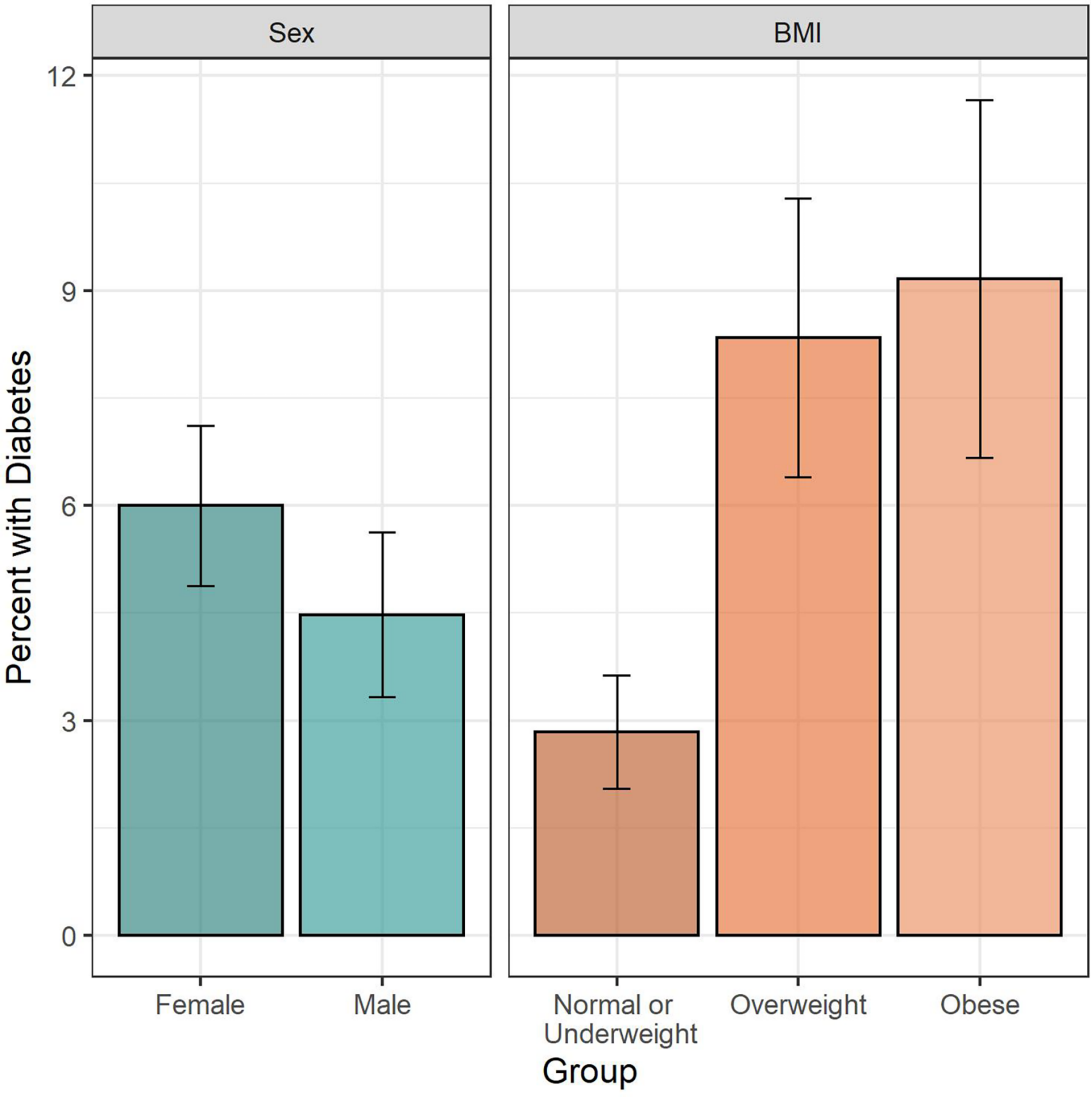
Diabetes prevalence by sex and BMI. Legend: Bar height indicates estimated diabetes prevalence, with 95% CI indicated by whiskers. Left panel shows diabetes prevalence by sex, right panel shows diabetes prevalence by BMI category.

Diabetes prevalence was highest among people between 50 to 59 years old, where it reached 11.1% compared to young participants aging 18 to 29 years old where it was under 1% (**Figure 1**). Diabetes prevalence in obese participants (9.2%) was 3-fold higher compared to people with underweight or normal BMI (2.8%) (**Figure 2**).

### Factors Associated With Diabetes in Multivariable Logistic Regression

In unadjusted analysis, advanced age was significantly associated with higher odds ratio of diabetes (60+ vs 18-29 years, OR 16.6, 95% CI 7.1 to 38.9) (**Table 3**). Higher BMI was also associated with higher odds of diabetes (obese vs underweight or normal, OR 3.4, 95% CI 2.3 to 5.2), as well as low physical activity (low vs moderate-high, OR 1.7, 95% CI 1.2 to 2.4).

**Table 3:** Factors associated with diabetes among adults in Haiti, multivariable logistic regression.

	Unadjusted ORs	Adjusted ORs
**Age, years**		
18-29	ref	ref
30-39	2.4 [0.84, 6.7]	2.3 [0.75, 7.0]
40-49	11.4 [4.8, 27.1]*	10.3 [3.9, 26.7]*
50-59	18.2 [7.8, 42.7]*	16.9 [6.5, 43.8]*
60+	16.6 [7.1, 38.9]*	17.7 [6.6, 47.9]*
**Male vs Female**	0.73 [0.53, 1.1]	1.1 [0.71, 1.6]
**Education**		
Primary or lower	ref	ref
Secondary or higher	0.46 [0.33, 0.63]*	1.4 [0.96, 2.2]
**Income (daily)**		
≤1 USD/day	0.9 [0.64, 1.3]	0.99 [0.69, 1.4]
>1 USD/day	ref	ref
**BMI, kg/m^2^**		
Underweight or Normal <24.9	ref	ref
Overweight 25.0-29.9	3.1 [2.1, 4.6]*	2.6 [1.7, 3.9]*
Obese ≥30.0	3.4 [2.3, 5.2]*	2.7 [1.7, 4.4]*
**Smoking status**		
Never/Former	ref	ref
Current	1.3 [0.57, 2.8]	1.4 [0.63, 3.2]
**Alcohol intake**		
≤1 drink a day	ref	ref
>1 drink a day	0.32 [0.08, 1.3]	0.6 [0.14, 2.6]
**Sugar sweetened beverage intake**		
0-3 days a week	ref	ref
4-7 days a week	0.45 [0.32, 0.61]*	0.56 [0.39, 0.78]*
**Physical activity**		
Low	1.7 [1.2, 2.4]*	1.3 [0.92, 1.8]
Moderate-high	ref	ref

*Indicates p value < 0.05.

Higher education was associated with a lower prevalence (secondary or higher vs primary or lower, OR 0.46, 95% CI 0.33 to 0.63). Interestingly, people who drank more sugar sweetened beverages had a lower odds of diabetes (4-7 days a week vs 0-3 days a week, OR 0.45, 95% CI 0.32 to 0.61).

In adjusted analysis, older age (60+ vs 18-29, OR 17.7, 95% CI 6.6 to 47.9) and higher BMI (obese vs normal or underweight, OR 2.7, 95% CI 1.7 to 4.4) remained statistically significantly associated with higher odds of having diabetes. Increased consumption of sugar sweetened beverages remained associated with a lower odds of diabetes (OR 0.56, 95% CI 0.39 to 0.78). This may be due to the fact that younger people are much more likely to have higher intake of sugar sweetened beverages, and also be less likely to have diabetes compared to older people. Further analysis on sugar sweetened beverage intake and age showed that those aged 18-29 years drank sugar sweetened beverages for a median of 6 days a week, compared to those aged 60+ who drank for a median of 4 days a week (chi square test p <0.001).

## Discussion

We found an age-adjusted prevalence of diabetes at 5.2% in a population-based cohort of adults in Port-au-Prince, Haiti. Factors associated with diabetes included increasing age, increasing BMI, and low physical activity.

The prevalence of diabetes in our sample in Haiti was relatively low compared to other LMICs, but similar to other Caribbean countries. In one survey using data from 28 LMICs, the reported prevalence of diabetes was 8.8% (11). Older studies from 1999 among the general population in Barbados and Jamaica report prevalence of 19.4% and 10.6%, respectively (12, 13). More recent estimates from pooled estimates in Barbados, Jamaica, and St Lucia report a diabetes prevalence of 7.2% (14). Notably, there are higher rates of diabetes among Black Caribbean populations who are immigrants in high income countries, such as 12.4% among Black Caribbean people in the Netherlands, and 9.5% in Canada (15). In Haiti, prior studies that measure blood glucose levels to report on diabetes prevalence are heterogeneous. A 2002 survey in Port au Prince reported an age standardized prevalence of 4.8% in men and 8.9% in women for diabetes, and 6.4% in men and 8.0% in women for prediabetes (6). Combining one rural (Cabral of Thomonde) and one urban (Riviere Froide, Carrefour) region of Haiti, one study reported a crude prevalence of diabetes at 23.1% in the rural region and 16.4% in the urban region, however there was a high proportion of those with chronic kidney disease in the sample (12.3%) which may indicate results are not generalizable outside the study population (16). Similar to these prior studies, we were not able to distinguish between type I and type II DM in our study sample, although the large majority were likely type II given only 6 participants were on insulin. It is possible that many people with type I DM do not survive past childhood in Haiti given the cost associated with strict insulin regimens.

The reason for low diabetes prevalence in Haiti may be related to the relatively low prevalence of obesity. We found only 17.2% of our sample had obesity, compared to 42.4% in the US (17). There also may be a genetic or epigenetic predisposition for lower diabetes in Black Caribbean populations compared to other people of African descent, like African Americans, related to either biologic drivers or social determinants of disease like systemic racism. Within each BMI category, our prevalence of diabetes (normal or underweight: 2.8%, overweight: 8.3%, obese: 9.2%) was lower than what has been reported for African Americans (normal: 13.5%, overweight: 17.8%, obese: 36.6%) (18). Black Caribbean populations have lower visceral and hepatic fat accumulation, and distinct hyper-insulinemic response to glucose stimulation (19), compared to Africans and African Americans, which may contribute to the lower prevalence of diabetes.

We found diabetes was more common in women versus men similar to prior literature, although this was not statistically significant. One systematic review on the distribution of diabetes in the Caribbean showed women were more likely to have diabetes (adjusted OR 1.65), obesity (adjusted OR 3.1), and be less physically active compared to men (20). There is a long-held belief in the Caribbean that obesity is healthy, and that larger, full-bodied women are associated with health and wealth (21). Furthermore, women are often told to “eat for the baby” during pregnancy, sometimes resulting in a higher BMI that does not decrease after birth (21). Furthermore, in Haiti abdominal obesity is not typically considered to be a medical problem because it signals health and prosperity, which may lead to increased risk for diabetes in the long-term.

The factors we found associated with a higher odds of diabetes, including age, increasing BMI, and lower physical activity, are consistent with prior studies (11, 16). While we were not able to test the association of fruit and vegetable intake on diabetes given the fact that everyone in our sample had uniformly low fruit and vegetable intake, prior studies in the Caribbean have shown that diets are high in fried fatty foods, partly because these diets are more cost-effective in terms of satiety (22, 23). There was a paradoxical association of higher sugar sweetened beverage intake associated with decreased odds of having diabetes, potentially due to younger adults having a higher intake of sugar sweetened beverages but lower prevalence of diabetes. In addition, we did not account for volume of sugar sweetened beverage intake, and our dichotomous split into beverage intake on 0-3 days vs 4-7 days is not validated. Increasingly mechanized environments and social conditions like crime contribute to widespread physical inactivity and sedentary lifestyles (5). In Haiti, many people are afraid to leave their homes to do exercise outdoors due to protests and gang kidnappings, and there is a lack of green spaces or urban infrastructure for physical activity (24).

While the prevalence of diabetes is relatively low in Haiti, there remains a need to improve surveillance systems and strengthen health systems to diagnose, prevent, and treat diabetes. Using service provision assessment surveys by the Demographic Health Surveillance program, a prior analysis showed many health facilities in Haiti lacked essential equipment to treat both acute inpatient and outpatient diabetes, with 40% of hospitals having blood glucose testing equipment, 12% having insulin, and 76% having metformin or glibenclamide (25). In addition to improving physical and human resources, peer education programs and religious faith have been harnessed in other Caribbean and Latin American countries to build programs to detect and manage diabetes (23, 24).

Strengths of this study include using a population-based sampling frame of adults in Haiti, and direct measurement of blood glucose values, primarily fasting, to diagnose diabetes. Limitations include only one measurement of glucose taken to define diabetes, while clinical practice often uses two measurements. There was no laboratory capacity to measure HgbA1c, and fasting status for labs was self-reported by patients. The original Haiti CVD Cohort excluded people with serious medical conditions, which may have resulted in selection bias against DM. Finally, we were only able to calculate prediabetes among people with fasting plasma glucose values, as the WHO does not have plasma glucose values for nonfasting labs, likely resulting in under-diagnosis of prediabetes.

In summary, we found a low prevalence of diabetes in adults living in Haiti. There was a higher odds of diabetes in those who were older, obese, and had low physical activity. There was also a trend of higher diabetes in women versus men, which may be due to cultural and societal preferences around higher BMI to signal health and wealth. While diabetes prevalence is low, there remains an important need to strengthen the Haitian health system to prevent, diagnose, and treat diabetes.

## Data Availability Statement

Deidentified data used for this analysis are available upon request after signing a data access and use agreement, provision of approval by the GHESKIO ethics board, and demonstration that the external investigative team is qualified and has documented evidence of human research protection training. Requests to access the datasets should be directed to liy9032@med.cornell.edu.

## Ethics Statement

This study involves human participants, and was reviewed and approved by the institutional review boards at Weill Cornell Medicine and Groupe Haitien d’Etude du Sarcome de Kaposi et des Infections Opportunistes (GHESKIO) (record number 1803019037). The patients/participants provided their written informed consent to participate in this study.

## Author Contributions

Conceived study: RS, MLM, LY. Data curation: RS, JLP, ED, VR, AA, SS, FP, JI, MM, OT, DN. Formal analysis: RS, LY. Funding acquisition: MLM. Investigation: RS, JLP, ED, VR. Methodology: RS, MG, MLM, LY. Project administration and resources: RS, JLP, ED, VR, RM, MD, JWP. Software: RS, LY. Writing-original draft preparation: RS, LY. Writing-review & editing: RS, JLP, ED, VR, AA, SS, FP, JI, MM, OT, DN, RM, MD, JWP, MG MLM, LY. All authors have read, and confirm that they meet ICMJE criteria for authorship. All authors contributed to the article and approved the submitted version.

## Funding

RS, JLP, ED, VR OT, JWP, MLM report a grant from NHLBI R01HL143788. MG reports a grant from NCI K08CA230318. MLM reports a grant from NHLBI D43TW011972, and Fogarty International Center R21TW011693.

## Conflict of Interest

RS, JLP, ED, VR OT, JWP, MLM report a grant from NHLBI Q23 R01HL143788. MLM reports a grant from NHLBI D43TW011972, and Fogarty International Center R21TW011693. The funders were not involved in the study design, collection, analysis, interpretation of data, the writing of this article or the decision to submit it for publication.

The remaining authors declare that the research was conducted in the absence of any commercial or financial relationships that could be construed as a potential conflict of interest.

## Publisher’s Note

All claims expressed in this article are solely those of the authors and do not necessarily represent those of their affiliated organizations, or those of the publisher, the editors and the reviewers. Any product that may be evaluated in this article, or claim that may be made by its manufacturer, is not guaranteed or endorsed by the publisher.
