# Supplementary material for: Diabetes Epidemiology Among Adults in Port-au-Prince, Haiti: A Cross-Sectional Study
Source: Front Endocrinol (Lausanne). 2022 Feb 24;13:841675. doi: 10.3389/fendo.2022.841675 (PMC8913034; doi:10.3389/fendo.2022.841675)
Supplement: Supplementary file 1 [file Image_1.pdf]

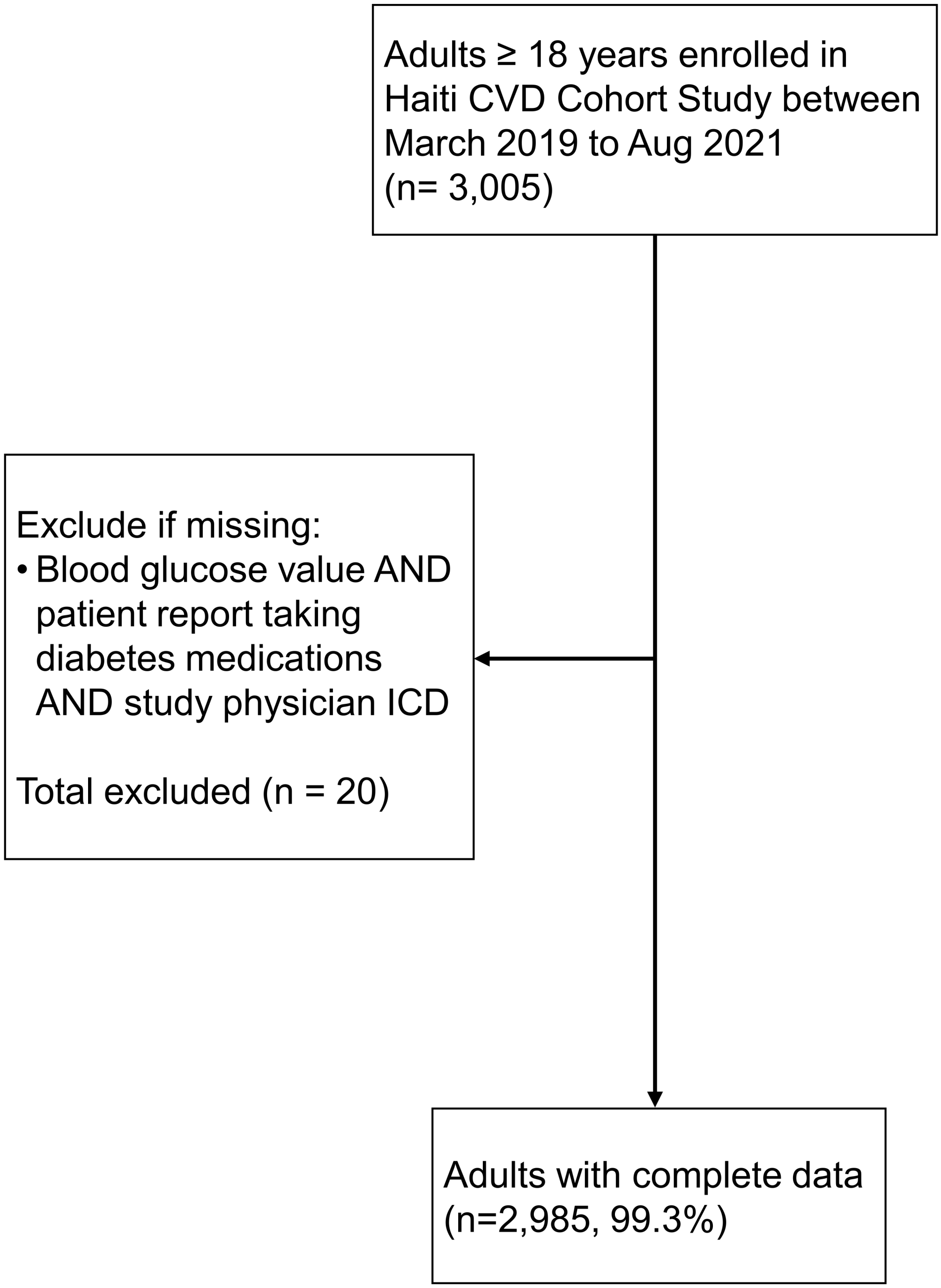

Adults ≥ 18 years enrolled in Haiti CVD Cohort Study between March 2019 to Aug 2021 (n= 3,005)
Exclude if missing:
• Blood glucose value AND patient report taking diabetes medications AND study physician ICD
Total excluded (n = 20)
Adults with complete data (n=2,985, 99.3%)
